# Investigating the causal role of immune cells in preeclampsia: Insights from Mendelian randomization analysis

**DOI:** 10.1097/MD.0000000000047713

**Published:** 2026-05-15

**Authors:** Li Ma, Lingyu Yao, Mengyao Jiang

**Affiliations:** a Department of Obstetrics and Gynecology, The Second Affiliated Hospital of Zhejiang Chinese Medical University, Hangzhou, China; b Department of Obstetrics and Gynecology, Tongde Hospital of Zhejiang Province (Zhijiang Branch District), Hangzhou, China.

**Keywords:** causal inference, immunity, MR analysis, placenta, preeclampsia, sensitivity analysis

## Abstract

Immune-related inflammation is linked to preeclampsia (PE), but immune cell–PE associations remain inconsistent. This study used two-sample Mendelian randomization (MR) to explore causal links between immune cell profiles and PE, informing clinical research and interventions. Genome-wide association study data included 731 immune cell phenotypes (3757 Europeans) and PE (2355 cases/264,887 controls, European descent). Single nucleotide polymorphisms were selected as instrumental variables (IVs) after quality control. Multiple MR methods (inverse variance weighted, weighted median estimator, weighted mode, and MR-Egger) and sensitivity analyses (heterogeneity, leave-one-out, and pleiotropy) were applied; false discovery rate (FDR) correction adjusted for multiple comparisons. Bidirectional MR explored reverse causality. After FDR correction (P_FDR < 0.20), 6 immunophenotypes showed significant causal associations with PE: 3 protective (CD62L^−^CD86^+^ myeloid dendritic cell [DC], CD62L^−^ myeloid DC, granulocyte SSC-A levels) and 3 risk-increasing (CD16 on CD14^+^CD16^+^ monocytes, human leukocyte antigens DR^+^ natural killer cells, programmed death-ligand 1 on CD14^−^CD16^+^ monocytes). No horizontal pleiotropy was detected, and results were robust. Reverse MR identified 18 suggestive immunophenotypes (*P* < 5 × 10^−6^) potentially affected by PE (predominantly B cells) but no reverse causality for the 6 key phenotypes. Six immune cell phenotypes have causal links to PE, highlighting monocytes, myeloid DCs, natural killer cells, and granulocytes in PE pathogenesis. These findings offer potential immune biomarkers and therapeutic targets, emphasizing the need for translational research to validate clinical utility.

## 1. Introduction

Preeclampsia (PE) is a complex systemic pregnancy complication characterized by the new onset of hypertension after 20 weeks of gestation, accompanied by 1 or more adverse outcomes such as proteinuria, maternal organ dysfunction, or uteroplacental insufficiency (e.g., fetal growth restriction, abnormal angiogenesis).^[1,2]^ As a leading cause of maternal and neonatal morbidity and mortality worldwide, PE affects approximately 4 million women annually, resulting in over 70,000 maternal deaths and 500,000 infant fatalities each year.^[1]^ Beyond the perinatal period, survivors of PE face long-term health risks, including increased susceptibility to stroke, cardiovascular diseases, and diabetes, while offspring are at higher risk of preterm birth, perinatal death, neurodevelopmental delays, and later-life metabolic and cardiovascular disorders.^[3–5]^ It is estimated that over 300 million women and children globally are at elevated risk of chronic health issues due to prior PE exposure, underscoring the urgent need to elucidate its underlying mechanisms and identify actionable targets for prevention and intervention.

Accumulating epidemiological and experimental evidence supports the notion that PE is an immune-mediated disease, characterized by dysregulation of both innate and adaptive immune responses.^[6–8]^ The pathogenesis of PE is closely linked to maternal immune imbalance at the maternal–fetal interface, where immune cells play critical roles in mediating tolerance to the semi-allogeneic fetus, regulating trophoblast invasion, and maintaining placental vascular integrity.^[9,10]^ Key immune cells involved in PE pathogenesis include natural killer (NK) cells, human leukocyte antigens (HLA), and T lymphocytes.^[11]^ NK cells secrete factors such as vascular endothelial growth factor, interleukins, and γ-interferon, which are essential for trophoblast invasion and placental angiogenesis; impaired decidual NK cell function can lead to inadequate trophoblast invasion and failed spiral artery remodeling, a hallmark of PE.^[12]^ HLA-C, expressed by extravillous trophoblasts, establishes protective maternal antibodies and shields trophoblasts from decidual NK cell lysis, while dysregulated HLA expression is associated with PE susceptibility.^[11]^ Decidual regulatory T cells (Treg), derived from immature CD4^+^ T cells, play a pivotal role in maintaining immune tolerance; reduced Treg numbers or dysfunction, accompanied by altered secretion of transforming growth factor-β1, has been linked to PE development.^[12]^

Despite these advances, studies investigating the association between immune inflammation and PE have yielded inconsistent results, attributed to limitations such as small sample sizes, methodological heterogeneities, and unaccounted confounding variables.^[13,14]^ Observational studies are further constrained by potential reverse causality and residual confounding, making it challenging to establish definitive causal relationships between immune cell phenotypes and PE.^[15,16]^ Mendelian randomization (MR) is an emerging genetic epidemiological method that uses genetic variants as instrumental variables (IVs) to infer causal relationships between exposures and outcomes.^[17]^ By leveraging the random assortment of genetic variants at conception, MR minimizes confounding and reverse causality, offering a robust approach to investigate causal links in observational data. Large-scale MR studies of immune cells have successfully identified disease biomarkers and elucidated biological pathways, making it a powerful tool for exploring the immune basis of complex diseases like PE.^[17]^

In this study, we employed a two-sample MR framework to evaluate the causal relationships between 731 immune cell phenotypes and PE, utilizing publicly available genome-wide association study (GWAS) data. We aimed to: identify immune cell phenotypes with causal effects on PE risk; explore potential reverse causality (i.e., whether PE affects immune cell phenotypes); validate the robustness of causal associations through comprehensive sensitivity analyses; and provide novel insights into the immune mechanisms underlying PE, with implications for clinical translation.

## 2. Materials and methods

### 2.1. Research design

In this study, we set out to explore the causal dynamics between PE and specific immune cell signatures, employing a robust two-sample MR framework. The causal inference was drawn from an analysis of single nucleotide polymorphisms (SNPs) associated with 731 immune cell profiles and PE, utilizing these genetic variants as instrumental variables to elucidate the relationship between the immune cell profiles (as exposure factors) and PE (as the outcome). To affirm the credibility of our outcomes, we engaged in a comprehensive validation process, including heterogeneity testing, sensitivity analyses, and multiplicity corrections, to minimize biases in our results. The foundation of Mendelian randomization in our study rests on 3 critical assumptions: a significant association between the instrumental variables and the exposure factors, the independence of instrumental variables from any confounders affecting the outcome, and the instrumental variables’ exclusive effect on the outcome through their interaction with the exposure factors.

### 2.2. Ethical review statement

This study utilized publicly available GWAS summary data (immune cell phenotypes: GCST0001391–GCST0002121; PE: ebi-a-GCST90018906) that were previously approved by the respective institutional review boards (IRBs) of the original study cohorts. The original immune cell GWAS was approved by the IRB of the University of Sassari (Sassari, Italy), and the PE GWAS was approved by the IRB of the MRC Integrative Epidemiology Unit (Bristol, UK). Informed consent was obtained from all participants in the original studies, as documented in their respective publications. Since this study involved secondary analysis of de-identified public data, no additional ethical approval or informed consent was required.

### 2.3. Immune scope GWAS data source

For analyzing GWAS data, we opted for peripheral blood immunophenotyping, accessing summary statistics for 731 immune traits from the GWAS catalog database (with accession numbers spanning GCST0001391–GCST0002121).^[18]^ This analysis, targeting a cohort of 3757 European adults and adjusted for sex and age, scrutinized approximately 22 million SNPs.^[19]^ The immune cell types analyzed included 118 absolute cell counts, 389 measures of median fluorescence intensity, 32 morphological parameters, and 192 relative cell counts, covering a spectrum of cell types such as B cells, dendritic cells (DCs), mature T cells, monocytes, myeloid cells, and TBNK (T cells, B cells, and NK cells), with morphological parameters features including panels for DC and TBNK cells.

### 2.4. Sources of GWAS data on preeclampsia

Our investigation sourced PE data (ID: ebi-a-GCST90018906) from the repository of GWAS summary statistics (https://gwas.mrcieu.ac.uk/). The dataset comprised 267,242 individuals of European descent, including 2355 PE patients and 264,887 control subjects. To reduce potential racial bias, all participants were genetically confirmed as European. The demographic specifics of this cohort are detailed in the study’s primary publication.

### 2.5. Instrumental variable (IV) selection

To validate the selected SNPs, we assessed linkage disequilibrium through clumping, setting a 5000 kb window size and an *r*^2^ threshold of <0.01 to isolate independent SNPs. The *F*-statistics were then computed to ascertain the robustness of each IV, eliminating weaker instruments through the formula:


$$\begin{array}{l} F=R^{2}\times (N-K-1)/[K\times (1-R^{2})], \end{array}$$

where *R*^2^ represents the variance in exposure explained by each IV, N is the GWAS sample size, and *K* the number of SNPs analyzed in the MR study. An *F*-value >10 was deemed indicative of sufficient instrument strength, thus minimizing the risk of bias from weak instruments. For this research, between 3 and 742 SNPs were identified as suitable IVs for immunophenotyping. SNPs for both exposure and outcome were harmonized based on alleles and their frequencies, adjusting for inconsistencies to ensure matching alleles.

### 2.6. Statistical methodology

To unravel the causal connection between immune cells and PE, we employed a variety of analytical techniques, including the inverse variance weighted (IVW) method, simple mode, MR-Egger regression, weighted mode (WM), and weighted median estimator.^[20]^ This array of methods allowed for a thorough investigation into the potential causal relationships. The IVW method, as the cornerstone for estimating causal effects in MR studies, is celebrated for its efficacy in uncovering causality with high testing precision.^[21]^ The MR-Egger approach introduces an intercept for pleiotropy assessment, mirroring the IVW method when the intercept nears zero. A notable shift from zero in this intercept suggests the presence of horizontal pleiotropy among the instrumental variables. The weighted median estimator technique offers unbiased estimations, even when up to half of the data might originate from less reliable instrumental variables.^[21]^ To counter the heightened risk of type I errors from multiple comparisons,^[22]^ we applied a FDR correction. Cochran *Q* statistic was pivotal for evaluating the heterogeneity among instrumental variables. “Leave-one-out” sensitivity analysis was meticulously performed, removing each SNP in turn to detect any disproportionately influential variants. Furthermore, scatter and funnel plots were instrumental in affirming the robustness and consistency of our findings, showcasing the stability and uniformity of the analysis. Additionally, a reverse MR analysis on immune cells identified from the initial MR provided further insight into the bidirectional causality between immune cells and PE, enhancing our understanding of the complex. A flow diagram summarizing the study design and analytical workflow of the two-sample Mendelian randomization analysis is provided in Figure S1.

## 3. Results

### 3.1. Investigating the impact of immune cell phenotypes on preeclampsia

**Figure 1.**
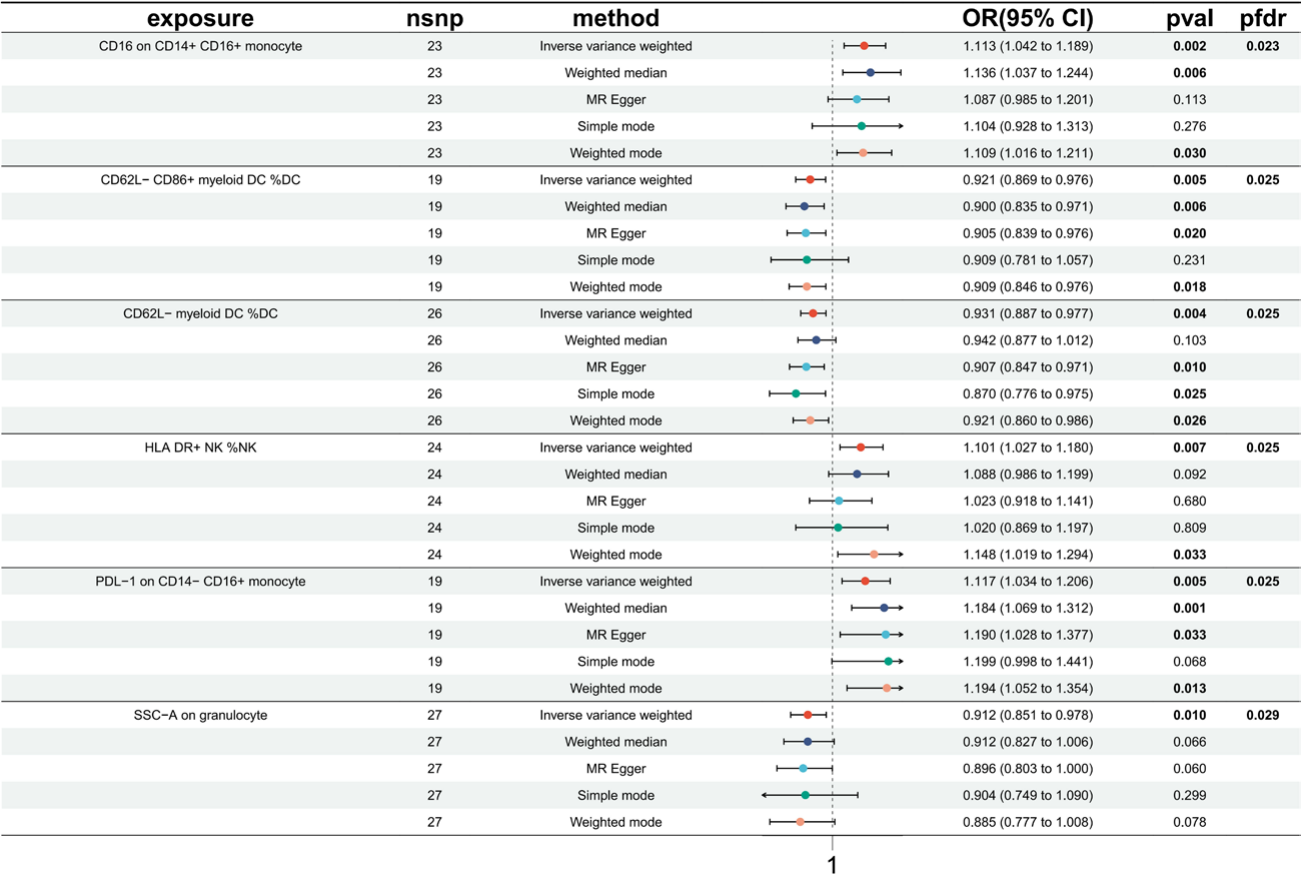
Forest plots showed the causal associations between immune cell traits and PE. CI = confidence interval; IVW = inverse variance weighting; PE = preeclampsia.

After applying a FDR correction (*P*_*FDR*_ < 0.20), our analysis highlighted 3 immunophenotypes that demonstrate protective effects against PE: *CD62L− CD86+ myeloid DC %DC* (myeloid cell panel), *CD62L− myeloid DC %DC* (myeloid cell panel), *SSC-A on granulocyte* (TBNK panel). Specifically, the odds ratio (OR) of *CD62L− CD86+ myeloid DC %DC* on PE risk was estimated to be 0.921 (95% CI = 0.869–0.976, *P* = .005, *P*_*FDR*_ = .025, Fig. 1, Tables S1 and S2) by using the IVW method. Similar results were observed by using 3 more methods: weighted median (OR = 0.900, 95% CI = 0.835–0.971, *P* = .006); MR Egger (OR = 0.905, 95% CI = 0.839–0.976, *P* = .020); and WM (OR = 0.909, 95%CI = 0.846–0.976, *P* = .018). The OR of *CD62L− myeloid DC %DC* on PE risk was estimated to be 0.931 (95% CI = 0.887–0.977, *P* = .004, *P*_*FDR*_ = .025, Fig. 1, Tables S1 and S2) by using the IVW method. Similar results were observed by using 3 more methods: MR Egger (OR = 0.907, 95% CI = 0.847–0.971, *P* = .010); simple mode (OR = 0.870, 95% CI = 0.776–0.975, *P* = .025); and WM (OR = 0.921, 95% CI = 0.860–0.986, *P* = .026). The OR of *SSC-A on granulocyte* on PE risk was estimated to be 0.912 (95% CI = 0.851–0.978, *P* = .01, *P*_*FDR*_ = .029, Fig. 1, Tables S1 and S2) by using the IVW method. Conversely, we also detected jeopardy effects of 3 immunophenotypes on preeclampsia: CD16 on CD14+ CD16+ monocyte (monocytes panel), HLA DR+ NK %NK (TBNK panel), programmed death-ligand 1 (PDL-1) on CD14− CD16+ monocyte (monocytes panel). Concretely, the OR of *CD16 on CD14+ CD16+ monocyte* on PE risk was estimated to be 1.113 (95% CI = 1.042–1.189, *P* = .002, *P*_*FDR*_ = .023, Fig. 1, Tables S1 and S2) by using the IVW method. Similar results were observed by using 2 more methods: weighted median (OR = 1.136, 95% CI = 1.037–1.244, *P* = .006), and WM (OR = 1.109, 95% CI = 1.016–1.211, *P* = .030). The OR of *HLA DR+ NK %NK* on PE risk was estimated to be 1.101 (95% CI = 1.027–1.180, *P* = .007, *P*_*FDR*_ = .025, Fig. 1, Tables S1 and S2) by using the IVW method. Similar results were observed by using WM (OR = 1.148, 95% CI = 1.019–1.294, *P* = .033). The OR of *PDL-1 on CD14− CD16+ monocyte* on PE risk was estimated to be 1.117 (95% CI = 1.034–1.206, *P* = .005, *P*_*FDR*_ = .025, Fig. 1, Tables S1 and S2) by using the IVW method. Similar results were observed by using 3 more methods: weighted median (OR = 1.184, 95% CI = 1.069–1.312, *P* = .001); MR Egger (OR = 1.199, 95% CI = 1.028–1.377, *P* = .033); WM (OR = 1.194, 95% CI = 1.052–1.354, *P* = .013). The MR-Egger intercept negated the possibility of horizontal pleiotropy across these links. Sensitivity analyses (Table S3), including scatter and funnel plots, underscored the robustness and consistency of these causal associations (Figs. S2 and S3).

### 3.2. Investigating preeclampsia’s causal impact on immune cell phenotypes

**Figure 2.**
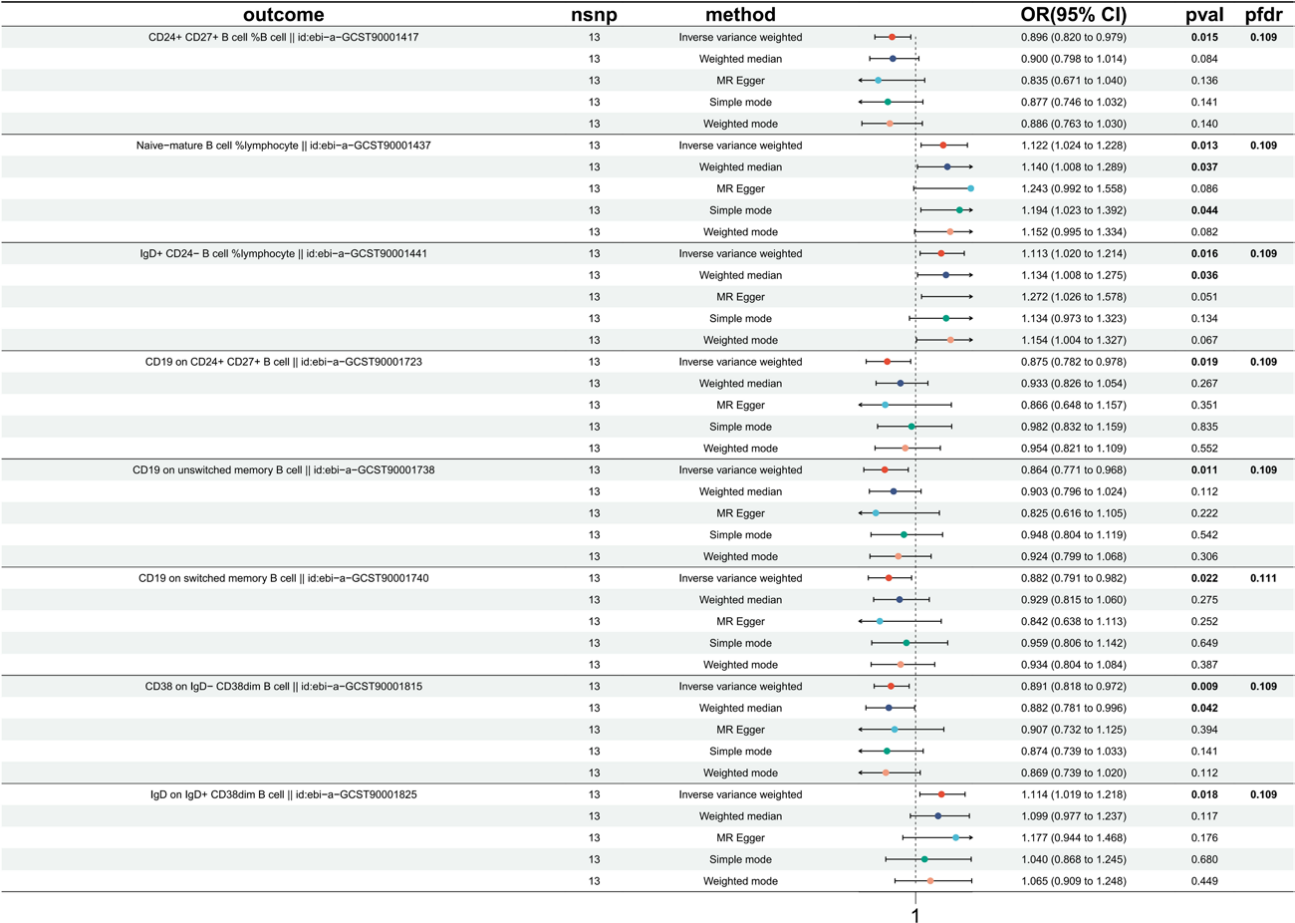
Forest plots showed the causal associations between PE and immune cell traits by using different methods. CI = confidence interval; IVW = inverse variance weighting; PE = preeclampsia.

Our two-sample MR analysis aimed to decipher the causal impact of PE on various immunophenotypes, with the IVW method serving as the principal analytic tool. While no immune trait reached statistical significance at *P* < 5 × 10E‐8, 18 suggestive immunophenotypes were identified at a significance level of *P* < 5 × 10E‐6, focusing on those with *P* < .03 as illustrated in Figure 2, predominantly within the B cell panel. We found that PE onset could increase the level of *Naive-mature B cell %lymphocyte* (β = 0.115, 95% CI = 1.024–1.228, *P* = .013, *P*_*FDR*_ = .109, Fig. 2). *IgD+ CD24− B cell %lymphocyte* were increased in PE patients (β = 0.107, 95% CI = 1.020–1.214, *P* = .016, *P*_*FDR*_ = .109, Fig. 2). *IgD on IgD+ CD38dim B cell* was also found to be increased (β = 0.108, 95% CI = 1.019–1.218, *P* = .018, *P*_*FDR*_ = .109, Fig. 2). The causal effect of PE on *CD24+ CD27+ B cell %B cell* was estimated to be ‐0.110 (95% CI = 0.896–0.979, *P* = .015, *P*_*FDR*_ = .109, Fig. 2), but the weighted median did not support this association: weighted median (β = ‐0.106, 95% CI = 0.798–1.014, *P* = .084). For *CD19 on CD24+ CD27+ B cell*, a negative association was observed (β = ‐0.134, 95% CI = 0.782–0.978, *P* = .019, *P*_*FDR*_ = .109, Fig. 2), which was inconsistent with weighted the mode. Similar associations were found for *CD19 on unswitched memory B cell* (β = ‐0.146, 95% CI = 0.771–0.968, *P* = .011, *P*_*FDR*_ = .109, Table S2), *CD19 on switched memory B cell* (β = ‐0.126, 95% CI = 0.791–0.982, *P* = .002, *P*_*FDR*_ = .111, Fig. 2), and *CD38 on IgD− CD38dim B cell* (β = ‐0.115, 95% CI = 0.818–0.972, *P* = .009, *P*_*FDR*_ = .109, Fig. 2). With additional methodologies and sensitivity analyses reinforcing the reliability of these observed causal relationships. Specifically, the MR-Egger intercept conclusively dismissed the notion of horizontal pleiotropy, with scatter and funnel plots further attesting to the stability and coherence of our findings (Figs. S4 and S5).

## 4. Discussion

This study is the first to systematically evaluate the causal relationships between 731 immune cell phenotypes and PE using a two-sample MR approach, leveraging large-scale GWAS data. Our findings identify 6 immunophenotypes with significant causal links to PE (3 protective, 3 risk-related) and 18 suggestive immunophenotypes potentially affected by PE, providing novel insights into the immune mechanisms underlying PE pathogenesis.

### 4.1. Monocyte subsets and PE risk

Monocytes, key components of the innate immune system, are classified into classical (CD14^+^ CD16^−^), intermediate (CD14^+^ CD16^+^), and nonclassical (CD14^−^ CD16^+^) subsets based on CD14 and CD16 expression, each with distinct roles in inflammation and tissue homeostasis.^[23]^ Our study found that increased CD16 levels on CD14^+^ CD16^+^ (intermediate) monocytes and PDL-1 expression on CD14^−^ CD16^+^ (nonclassical) monocytes are causally associated with elevated PE risk. This aligns with previous observational studies showing abnormal monocyte subset distribution in PE patients, characterized by increased intermediate and nonclassical monocytes compared to healthy pregnancies.^[24–26]^ Intermediate monocytes are considered a transitional subset in monocyte differentiation, while nonclassical monocytes are involved in endothelial repair,^[27]^ suggesting a potential compensatory response to endothelial damage in PE. However, our MR results indicate that elevated levels of these subsets are not merely a consequence of PE but a causal risk factor, highlighting their role in initiating or exacerbating PE-related pathogenesis.

Programmed death-ligand 1 is an immune checkpoint molecule that modulates T cell responses and immune tolerance.^[28]^ Increased PDL-1 expression on nonclassical monocytes may disrupt maternal-fetal immune tolerance by inhibiting T cell activation, leading to inadequate trophoblast invasion and placental dysfunction.^[16]^ CD16 (FcγRIII) is a receptor involved in antibody-dependent cellular cytotoxicity and cytokine production^[29]^; elevated CD16 levels on intermediate monocytes may enhance pro-inflammatory cytokine secretion, contributing to the systemic inflammation observed in PE.^[7,8]^ These findings suggest that targeting monocyte subset-specific markers (CD16, PDL-1) could be a potential therapeutic strategy for PE prevention or management.^[14]^

### 4.2. Myeloid DC subsets and protective effects against PE

Myeloid DCs are critical antigen-presenting cells that regulate immune responses by activating or tolerizing T cells.^[30,31]^ Our study identified 2 myeloid DC subsets (CD62L^−^, CD86^+^, and CD62L^−^) with causal protective effects against PE. CD62L (L-selectin) is involved in leukocyte homing and migration,^[23]^ while CD86 is a co-stimulatory molecule that promotes T cell activation.^[12]^ The CD62L^−^ phenotype indicates a mature, activated DC subset, and CD86 expression suggests enhanced capacity to prime T cells.^[32,33]^ The protective role of these DC subsets may be attributed to their ability to promote maternal-fetal immune tolerance by inducing Treg differentiation or suppressing pro-inflammatory T cell responses.^[16]^ Previous studies have shown that DC dysfunction is associated with PE, characterized by impaired antigen presentation and altered cytokine secretion.^[14]^ Our MR results confirm that these DC subsets are not just biomarkers but causally protective against PE, emphasizing the importance of DC-mediated immune regulation in maintaining a healthy pregnancy.^[8,15]^

### 4.3. NK cells and PE susceptibility

NK cells are the most abundant immune cells in the decidua, playing a key role in trophoblast invasion and placental vascular remodeling.^[34]^ Our study found that increased proportions of HLA DR^+^ NK cells within the total NK cell population are causally associated with elevated PE risk. HLA DR is a major histocompatibility complex class II molecule involved in antigen presentation^[11]^; HLA DR^+^ NK cells are considered an activated subset with enhanced cytotoxicity and cytokine secretion.^[34]^ Abnormal activation of NK cells may disrupt trophoblast function and spiral artery remodeling, leading to placental insufficiency.^[13]^ Previous studies have reported altered NK cell phenotype and function in PE patients, including increased cytotoxicity and pro-inflammatory cytokine production.^[32]^ Our MR results extend these findings by establishing a causal link between HLA DR^+^ NK cells and PE risk, suggesting that NK cell activation status is a critical determinant of PE susceptibility.^[14]^

### 4.4. Granulocyte SSC-A and PE protection

Granulocytes (neutrophils, eosinophils, and basophils) are key mediators of innate immunity and inflammation.^[15]^ SSC-A (side scatter area) is a flow cytometric parameter reflecting cell granularity, with higher SSC-A indicating increased granularity (e.g., mature neutrophils with more cytoplasmic granules).^[35]^ Our study found that higher SSC-A levels in granulocytes are causally associated with reduced PE risk. Granulocytes are involved in regulating inflammation and tissue repair^[35]^; increased granularity may indicate enhanced phagocytic capacity or altered cytokine secretion, contributing to immune homeostasis at the maternal–fetal interface.^[16]^ Previous studies have shown abnormal granulocyte activation in PE, characterized by increased neutrophil extracellular trap formation and pro-inflammatory cytokine release.^[15]^ Our findings suggest that granulocyte granularity (measured by SSC-A) may be a marker of functional competence, with higher granularity conferring protection against PE by promoting anti-inflammatory responses or tissue repair.^[14]^

### 4.5. Bidirectional MR: PE and suggestive immunophenotypes

Bidirectional MR analysis identified 18 suggestive immunophenotypes potentially affected by PE, predominantly in the B cell panel. PE was associated with increased naive-mature B cells, IgD^+^ CD24^−^ B cells, and IgD expression on IgD^+^ CD38dim B cells, and decreased CD19 expression on memory B cells and CD38 expression on IgD^−^ CD38dim B cells. B cells play a dual role in pregnancy: they produce protective antibodies against paternal antigens and regulate immune tolerance through cytokine secretion.^[36,37]^ Abnormal B cell responses, including increased autoantibody production and altered B cell subset distribution, have been observed in PE patients.^[38–40]^ Our suggestive findings indicate that PE may induce secondary changes in B cell phenotypes, potentially exacerbating immune imbalance.^[38,41]^ However, these associations did not reach strict statistical significance and were not consistently replicated across multiple MR methods, highlighting the need for further validation in independent cohorts.^[42]^ Notably, PE did not exert significant causal effects on the 6 key immunophenotypes identified in the forward MR analysis, confirming the directionality of causality (immune cell phenotypes → PE) for these markers.^[17]^

### 4.6. Clinical implications

Our findings have important clinical implications for PE prevention, diagnosis, and treatment. The 6 key immunophenotypes identified could serve as potential biomarkers for PE risk stratification.^[14]^ For example, measuring CD16 levels on intermediate monocytes, PDL-1 expression on nonclassical monocytes, and HLA DR^+^ NK cell proportions in early pregnancy may help identify women at high risk of PE, enabling targeted preventive interventions (e.g., anti-inflammatory therapies, immune modulators).^[10]^ Additionally, the protective DC subsets (CD62L^−^, CD86^+^, and CD62L^−^ myeloid DCs) and granulocyte SSC-A could be targeted for therapeutic activation to enhance maternal–fetal immune tolerance and reduce PE risk.^[16]^

Translational research is needed to validate these findings in clinical settings.^[14,15]^ For instance, longitudinal studies could assess whether these immune cell phenotypes predict PE onset in early pregnancy, and interventional studies could test whether modulating monocyte subset function (e.g., inhibiting CD16 or PDL-1) or enhancing DC subset activity reduces PE risk.^[24]^ Furthermore, the suggestive B cell phenotypes affected by PE could provide insights into long-term immune sequelae of PE, helping to explain the increased risk of chronic inflammatory diseases in PE survivors.

### 4.7. Limitations and future directions

Despite its strengths (large sample size, robust MR design, and comprehensive sensitivity analyses), this study has limitations. First, the GWAS data were limited to European populations, so the generalizability of our findings to other ethnicities is unclear.^[19]^ Genetic background and immune responses vary across ethnic groups, and future studies should include diverse populations to enhance the external validity of the results. Second, we used a relatively lenient FDR threshold (PFDR < 0.20) to capture potential causal associations, which may increase the risk of false positives.^[22]^ However, this threshold was justified to explore the understudied immune–PE relationship, and all significant associations were supported by multiple MR methods and sensitivity analyses, reducing the likelihood of spurious results.^[21]^ Third, the immune cell phenotypes were measured in peripheral blood, not at the maternal-fetal interface (e.g., decidua), where immune interactions are most relevant to PE.^[16]^ Future studies should utilize GWAS data on decidual immune cells to better reflect the local immune environment.^[33]^ Fourth, while MR minimizes confounding, it cannot account for gene–environment interactions, which may play a role in PE pathogenesis.^[42]^ Finally, the suggestive associations between PE and B cell phenotypes require validation in larger, independent cohorts to confirm their clinical relevance.^[38]^

Future research directions include: replicating the findings in non-European populations; investigating the molecular mechanisms underlying the causal associations (e.g., how CD16 on intermediate monocytes contributes to PE); developing clinical assays to measure the key immunophenotypes for PE risk assessment; conducting interventional studies to target the identified immune pathways; and integrating multi-omics data (genomics, transcriptomics, and proteomics) to gain a comprehensive understanding of the immune landscape of PE.

In conclusion, our two-sample MR study identifies 6 immune cell phenotypes with significant causal links to PE, highlighting the role of monocyte subsets, myeloid DC subsets, NK cells, and granulocytes in PE pathogenesis. These findings provide novel immune biomarkers and therapeutic targets for PE, advancing our understanding of the immune mechanisms underlying this devastating pregnancy complication.

## Author contributions

**Formal analysis:** Lingyu Yao.

**Writing – original draft:** Li Ma.

**Writing – review & editing:** Mengyao Jiang.
















