# Supplementary material for: Investigating the causal role of immune cells in preeclampsia: Insights from Mendelian randomization analysis
Source: Medicine (Baltimore). 2026 May 15;105(20):e47713. doi: 10.1097/MD.0000000000047713 (PMC13183093; doi:10.1097/MD.0000000000047713)
Supplement: Supplementary file 1 [file medi-105-e47713-s001.docx]

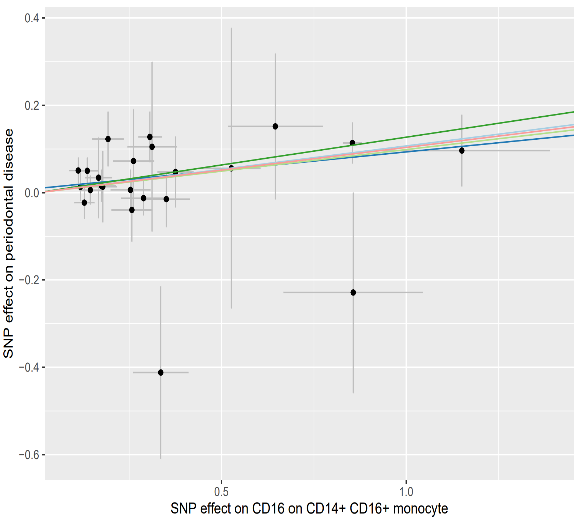

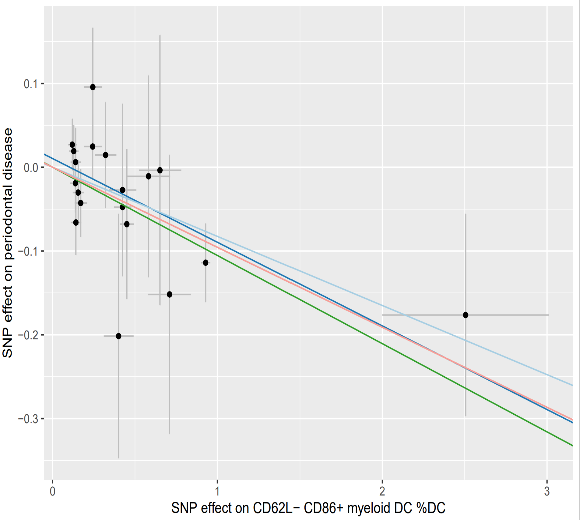

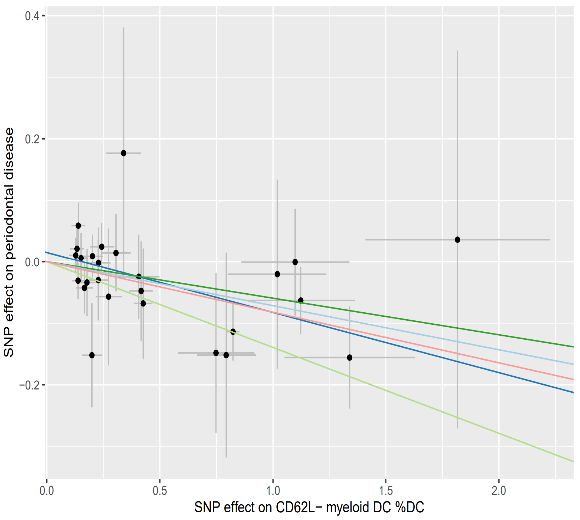

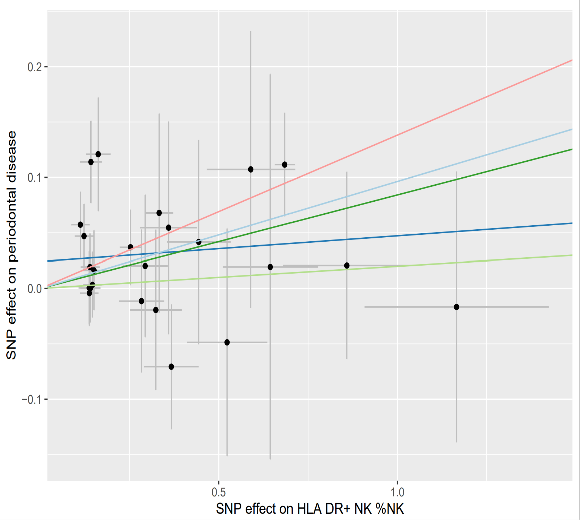

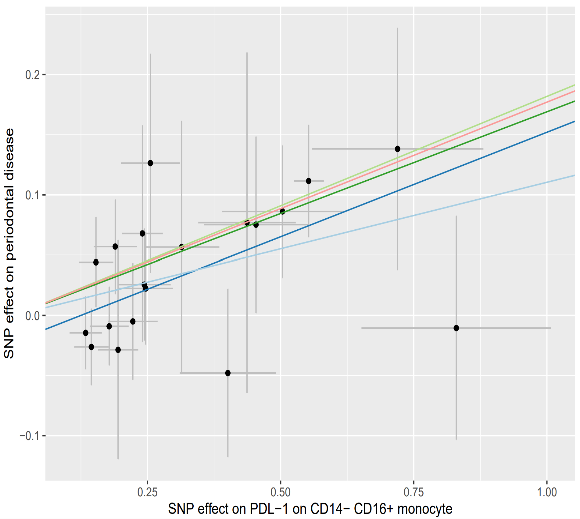

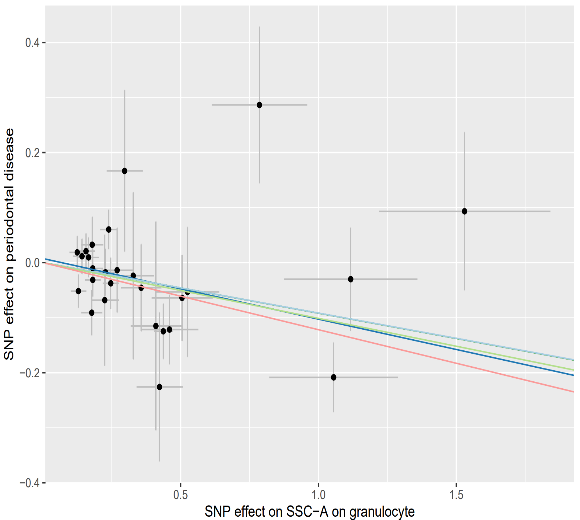


**Supplementary Figure 1.** Causal effects of immune cells concentration on PE. (A) Scatter plot between CD16 on CD14+ CD16+ monocyte and PE risk; (B) Scatter plot between CD62L- CD86+ myeloid DC _DC and PE risk; (C) Scatter plot between CD62L- myeloid DC _DC and PE risk; (D) Scatter plot between HLA DR+ NK _NK and PE risk; (E) Scatter plot between PDL-1 on CD14- CD16+ monocyte and PE risk; (F) Scatter plot between SSC-A on granulocyte and PE risk.
