# Supplementary material for: Investigating the causal role of immune cells in preeclampsia: Insights from Mendelian randomization analysis
Source: Medicine (Baltimore). 2026 May 15;105(20):e47713. doi: 10.1097/MD.0000000000047713 (PMC13183093; doi:10.1097/MD.0000000000047713)
Supplement: Supplementary file 4 [file medi-105-e47713-s004.docx]

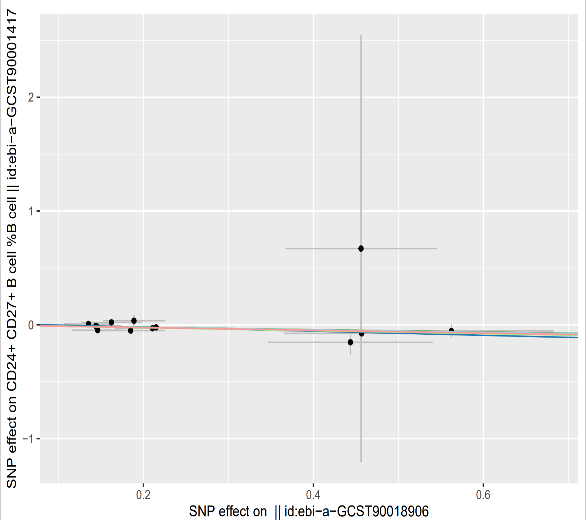

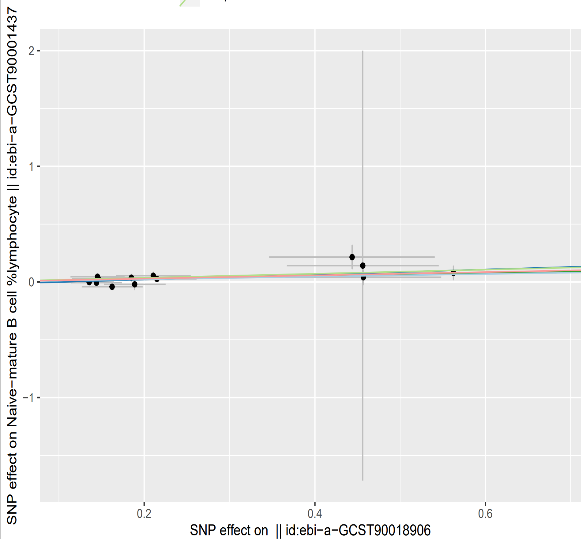

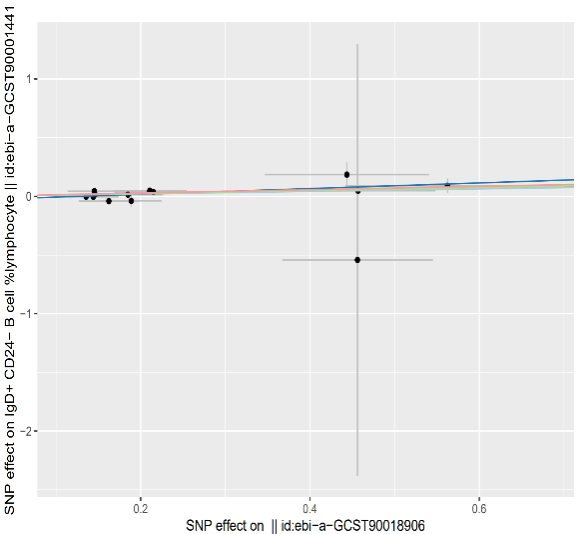

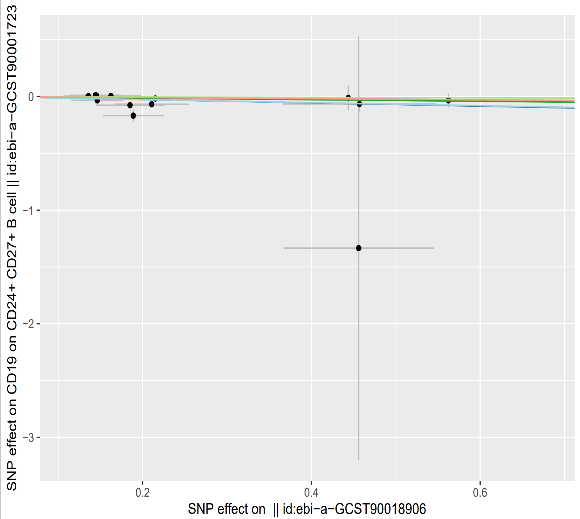

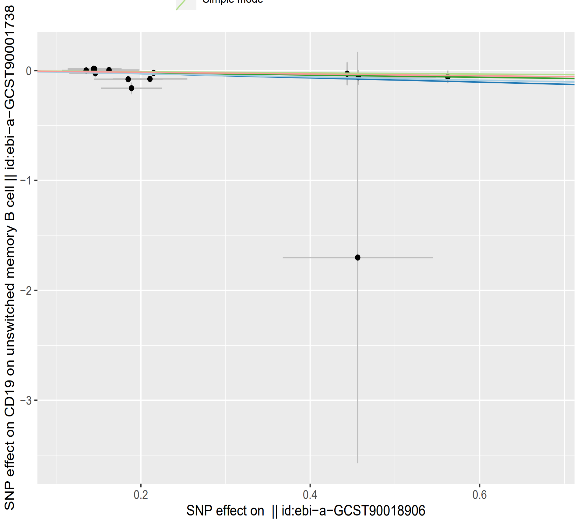

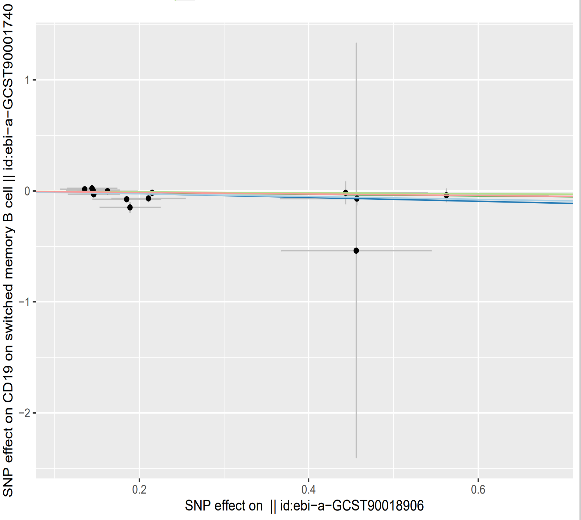


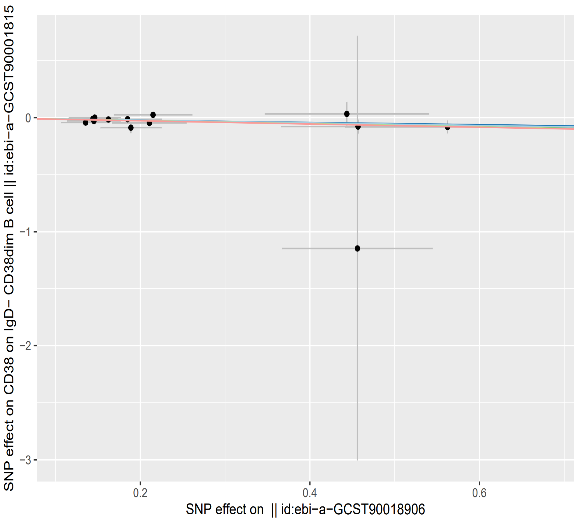

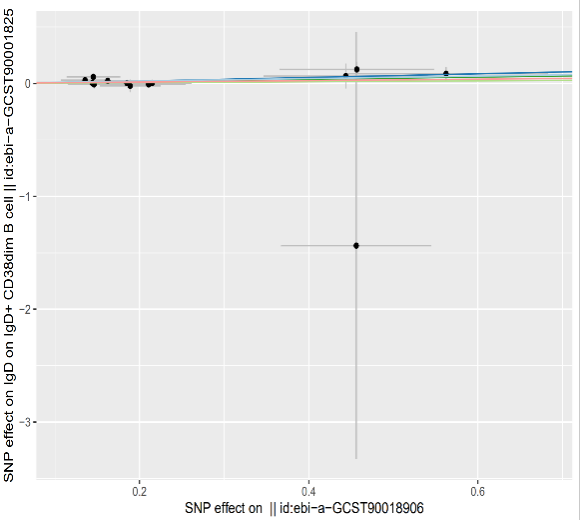


**Supplementary Figure 2.** Causal associations between PE and immune cells. (A) Scatter plot between Naïve CD24+ CD27+ B cell%B cell and PE risk; (B) Scatter plot between Naïve-mature B cell%lymphocyte and PE risk; (C) Scatter plot between IgD+ CD24- B cell %lymphocyte and PE risk; (D) Scatter plot between CD19 on CD24+ CD27+ B cell and PE risk; (E) Scatter plot between CD19 on unswitched memory B cell and PE risk; (F) Scatter plot between CD19 on switched memory B cell and PE risk; (G) Scatter plot between IgD+ CD38- B cell and PE risk.
