# Supplementary figures and images for: Investigating the causal role of immune cells in preeclampsia: Insights from Mendelian randomization analysis
Source: Medicine (Baltimore). 2026 May 15;105(20):e47713. doi: 10.1097/MD.0000000000047713 (PMC13183093; doi:10.1097/MD.0000000000047713)

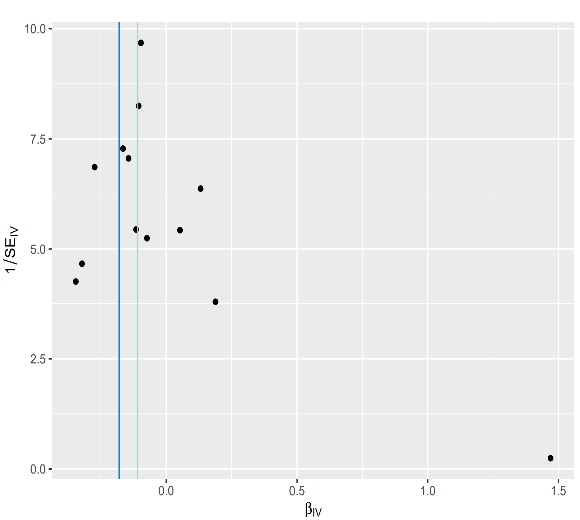

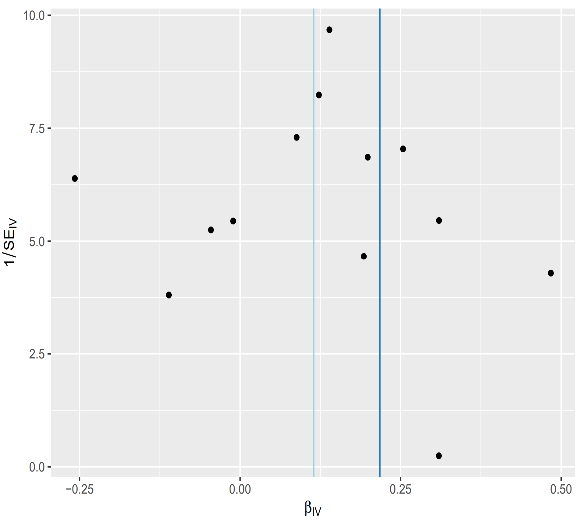

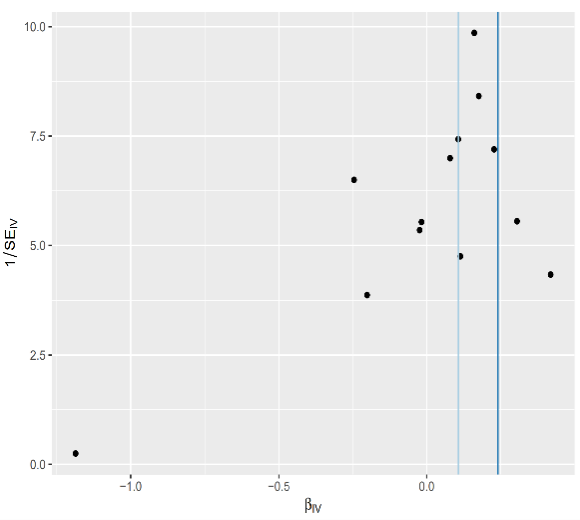

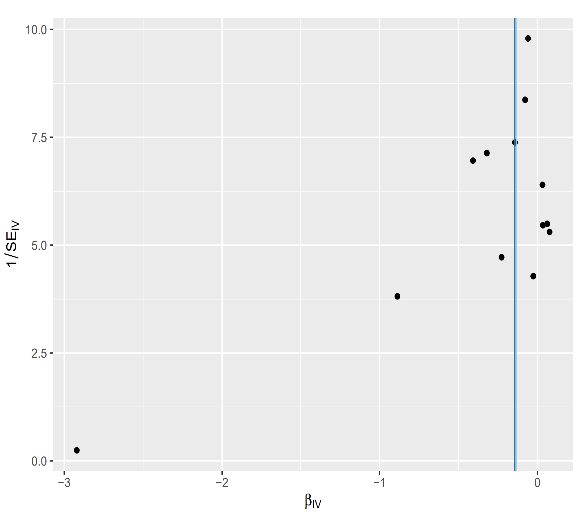

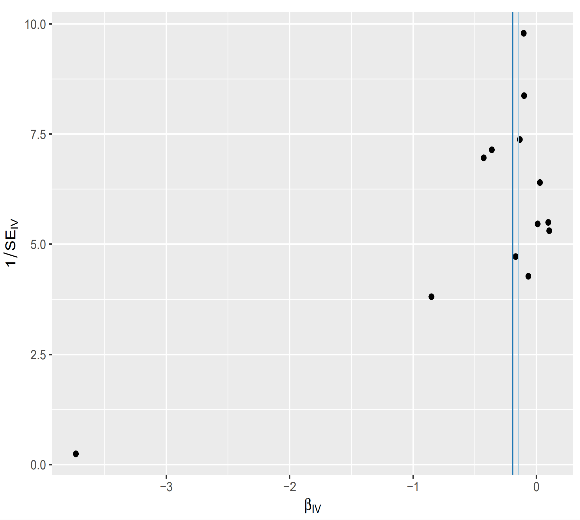

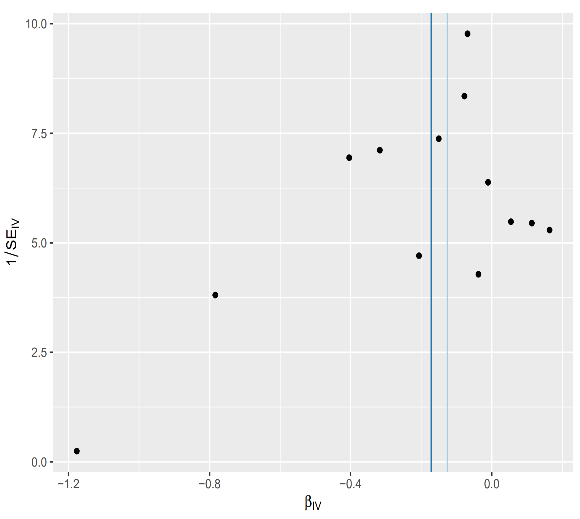


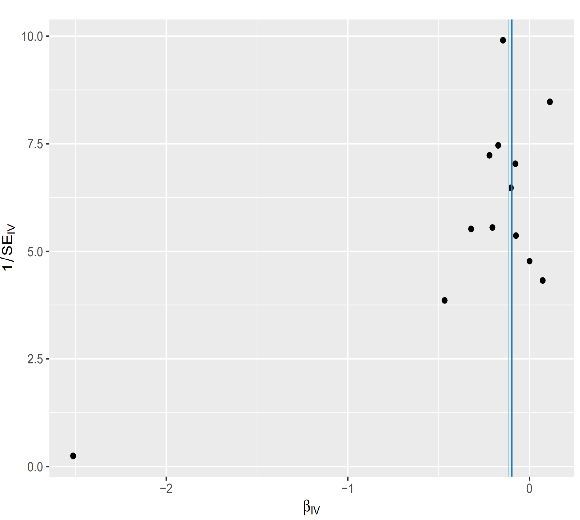

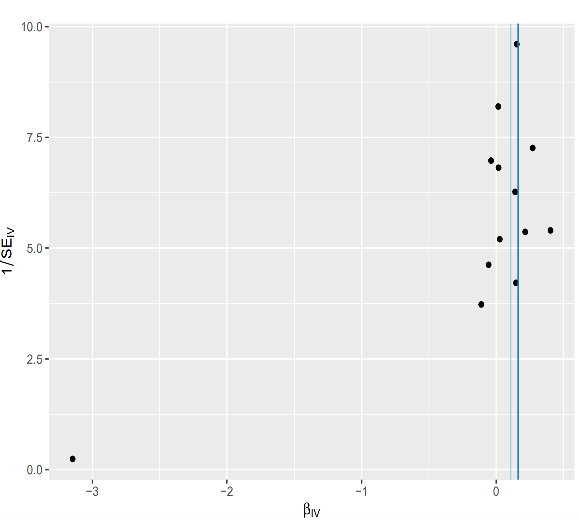


**Supplementary Figure 4.** Funnel plots between PE and immune cells.

Supplement: Supplementary file 6 [file medi-105-e47713-s006.docx]

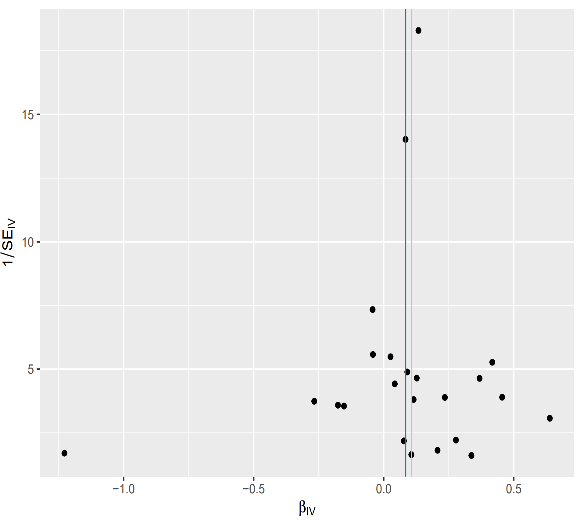

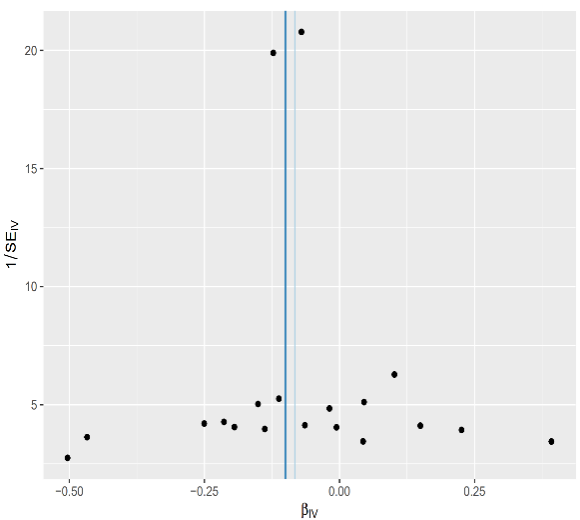

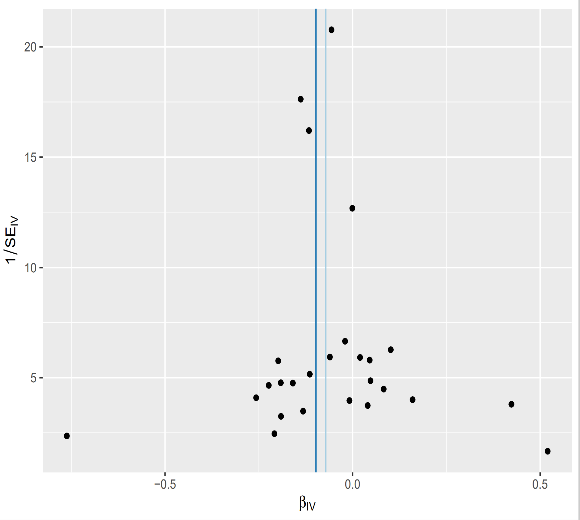

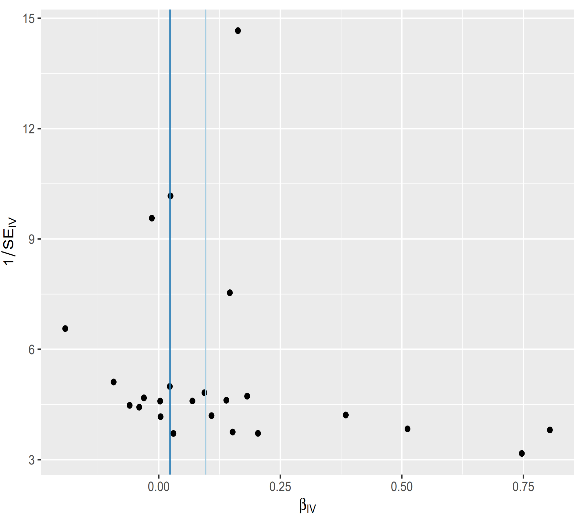

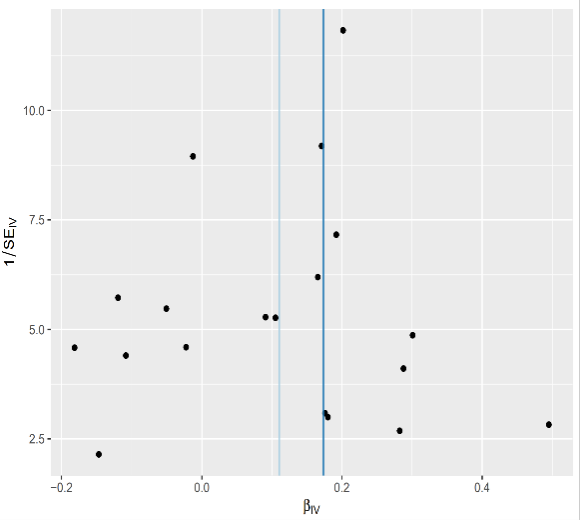

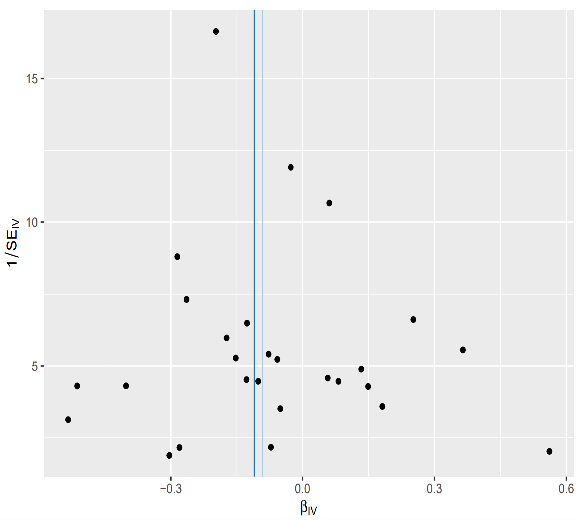


**Supplementary Figure 3.** Funnel plots between immune cells and PE.

Supplement: Supplementary file 7 [file medi-105-e47713-s007.docx]
