# Supplementary material for: Investigating the causal role of immune cells in preeclampsia: Insights from Mendelian randomization analysis
Source: Medicine (Baltimore). 2026 May 15;105(20):e47713. doi: 10.1097/MD.0000000000047713 (PMC13183093; doi:10.1097/MD.0000000000047713)
Supplement: Supplementary file 8 [file medi-105-e47713-s008.docx]

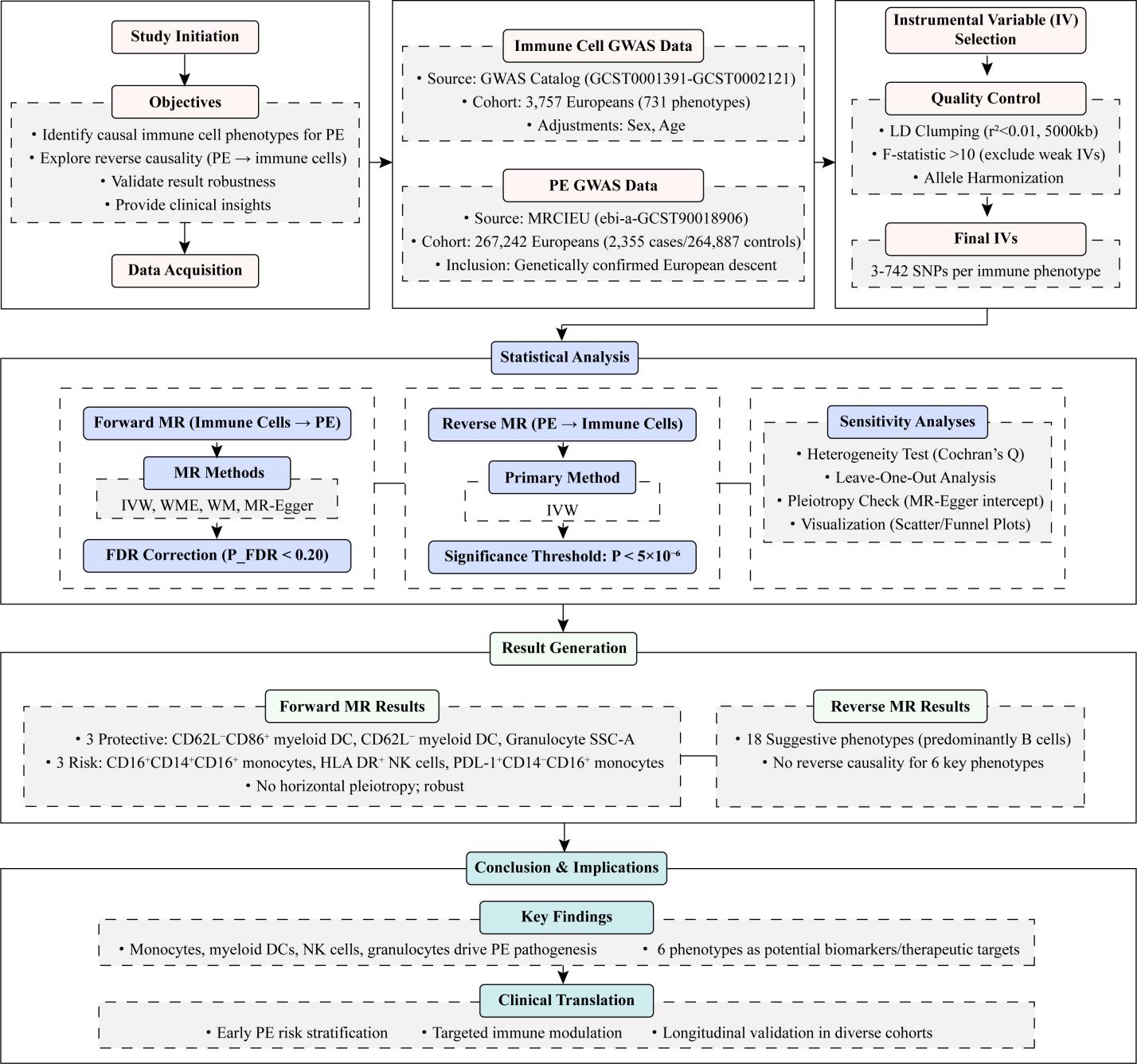


**Supplementary Figure 5.** Flow Diagram of the Mendelian Randomization Study on Immune Cells and PE
